# The impact of type 2 diabetes on adolescent and family functioning and healthcare use: findings from the National Survey of Children’s Health

**DOI:** 10.3389/fendo.2025.1589745

**Published:** 2025-06-16

**Authors:** Noël C. Slesinger Roy, Megan O. Bensignor, Wendy S. Grolnick, Peter B. Scal

**Affiliations:** ^1^ Division of Clinical Behavioral Neuroscience, Department of Pediatrics, University of Minnesota, Minneapolis, MN, United States; ^2^ Division of Pediatric Endocrinology, Department of Pediatrics, Center for Pediatric Obesity Medicine, University of Minnesota, Minneapolis, MN, United States; ^3^ Psychology Department, Clark University, Worcester, MA, United States; ^4^ Division of General Pediatrics and Adolescent Health, Department of Pediatrics, University of Minnesota, Minneapolis, MN, United States

**Keywords:** family functioning, adolescent health, type 2 diabetes, healthcare use, stress

## Abstract

**Introduction:**

Type 2 diabetes is a complex chronic illness requiring significant support. The goal of this study is to better understand stressors associated with type 2 diabetes for adolescents and caregivers and their impact on family functioning and health status.

**Methods:**

Using the National Survey of Children’s Health, this study examined differences in adolescent mental health, family and caregiver functioning, and medical service usage in pediatric type 2 diabetes, asthma, special healthcare needs, and adolescents without chronic illness.

**Results:**

Adolescents and families with type 2 diabetes demonstrated higher mental health concerns, poorer parental coping and family resilience, and higher use of emergency care than one or more of the comparison groups. Emergency care needs were also higher than expected for type 2 diabetes.

**Discussion:**

Adolescents with type 2 diabetes and their caregivers reported significant stressors and functioning concerns as compared to adolescents with and without another chronic illness. These families also required more intensive care needs, highlighting the need to support adolescent and family psychosocial functioning in the context of type 2 diabetes.

## Introduction

There has been a significant increase in pediatric type 2 diabetes, coinciding with the rise in pediatric obesity (1). The rate of type 2 diabetes is rising most amongst minority youth, particularly Latine and Non-Hispanic Black populations, which have been shown to present with higher hemoglobin A1c, comorbidities, and complications (1, 2). Youth with type 2 diabetes report lower healthcare-related quality of life (QOL) as compared to youth with type 1 diabetes or other chronic conditions.

In addition to low QOL and medical adherences, youth with type 2 diabetes demonstrate significant mental health concerns, including higher rates of depressive symptoms, difficulties with adherence and psychosocial functioning, and lower self-reported healthcare-related quality of life (HQoL) (3). In addition to the cognitive, behavioral, and social challenges related to understanding and managing a new chronic medical condition (St. 4), many youth with type 2 diabetes experience comorbid obesity, which is associated with weight-related external and internal stigma and bias (5). Stigma associated with obesity may also exacerbate challenges associated with type 2 diabetes, as those using insulin reported higher rates of diabetes stigma (6, 7).

Given these complexities, the American Diabetes Association (ADA) (8) recommends that all youth with type 2 diabetes receive comprehensive diabetes education and support, in addition to weight management programs that are developmentally and culturally appropriate as needed. Family lifestyle and behavior modifications are also an important component of intervention (9). For adults with type 2 diabetes, social support has been found to improve glycemic control and limit emotional distress (10). Family functioning and behavioral problems have also been found to impact adherence and glycemic control in type 1 diabetes (11). Despite the strong need for family-based lifestyle modifications in type 2 diabetes and the impact family functioning can have on pediatric obesity (12), little is known about the relation between family functioning and disease management in pediatric type 2 diabetes.

Understanding family, parent, and youth stressors and functioning, parent-child relationships, and the impact of these on youth physical and mental health may help to develop more comprehensive and effective interventions for youth with type 2 diabetes and their families.

Thus, using the National Survey of Children’s Health (NSCH) (13), this cross-sectional study examined the relationship between stressors, including parent and family functioning, physical, and mental health outcomes, in type 2 diabetes as compared to youth with other chronic illnesses, special healthcare needs (SHCN), and those without chronic illness.

## Method

Overview: This study is a secondary exploratory analysis of data from the 2022 NSCH, a household survey fielded annually for the purpose of examining the health and wellbeing, as well as their determinants, of children ages birth - 17 years in the United States. Beginning in 2022, the NSCH includes questions which identify children and adolescents with type 2 diabetes within the overall sample– providing a unique opportunity to pursue this research.

Sampling and Data Collection: The NSCH employs stratified sampling to create a sample capable of generating national and state level estimates. The questionnaire is completed in English or Spanish by a parent or knowledgeable adult and covers topics related to the randomly chosen child and the adult respondent, the household and neighborhood. Of the initial 358,000 households queried, 122,000 completed the screening; 67,269 households had children; and 54,103 completed the detailed questionnaire. The overall response rate is 39.1%.

Respondents were parents or caregivers of adolescents, categorized into one of four groups based on the adolescent’s health condition: 1) type 2 diabetes, 2) asthma, 3) special healthcare needs, or 4) no chronic condition. Categories were selected based on the availability of identifying questions and sample sizes.

**Figure 1:**
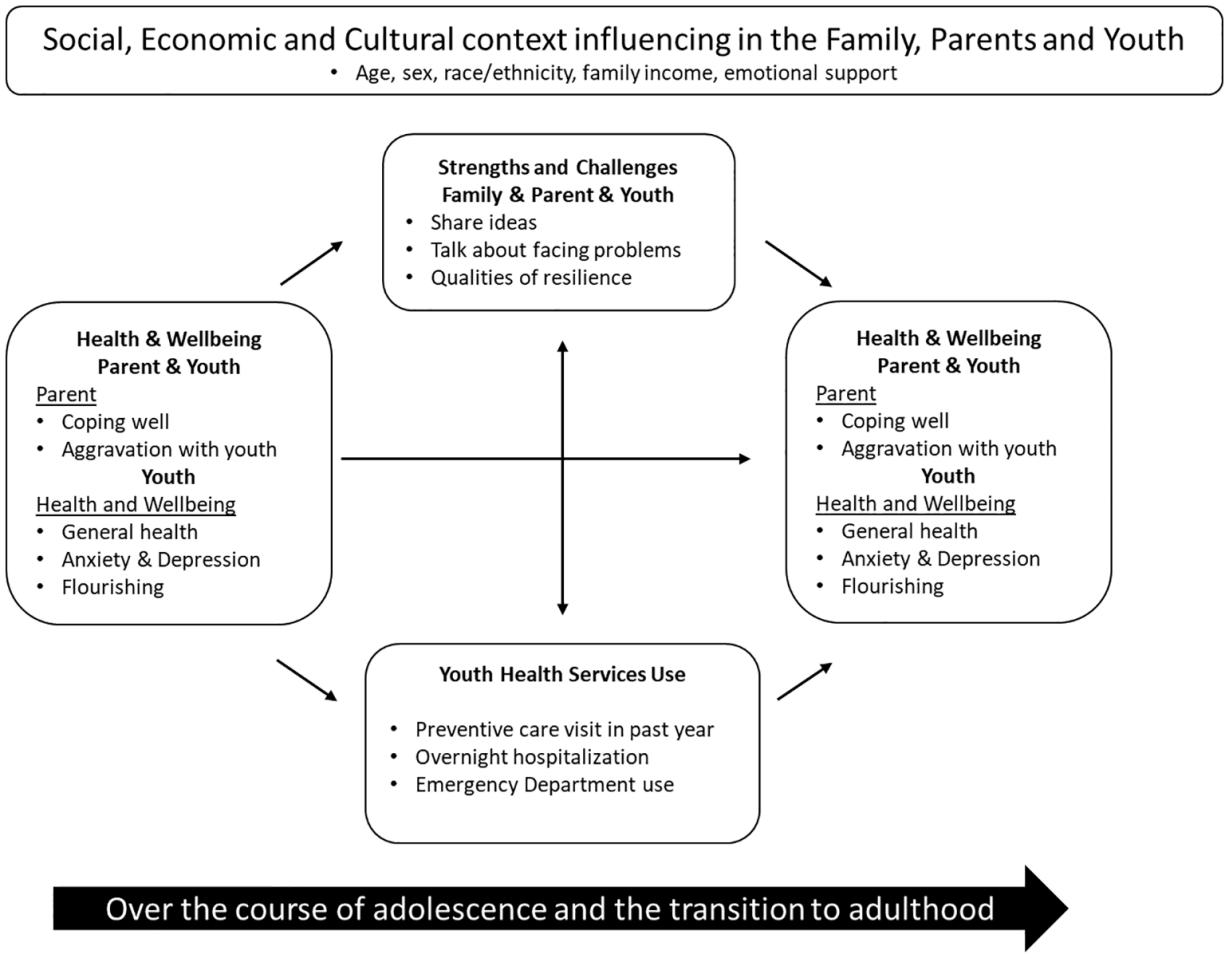
Conceptual Model Linking Family, Parent and Youth Health. The model depicts the bidirectional relations among strengths and challenges that the family and youth face, youth and parent coping and well-being, and healthcare service use during the transition from adolescence to adulthood.

Items selected for this analysis represent the stress, coping, and resilience paradigms that frame this analysis (**Figure 1**).

Data and Resource Availability: The dataset analyzed in the current study is available at the Data Resource Center for Child and Adolescent Health. The dataset includes both individual questions and aggregated constructs (i.e., parent aggravation) compiled by the distributors to assure consistency of use among analysts. P.B.S. acquired the dataset and takes responsibility for the accuracy of the data analysis.

Analysis: For descriptive analysis, unweighted frequencies are reported. All other values and statistical tests are calculated using the NCHS provided sample weights and survey design variables to allow for generalization to the US population and adjusting for standard errors. Differences between the type 2 diabetes cohort and each other cohort were tested individually using a parametric Wald test, which evaluates model-based estimates under the specified distributional assumptions of the regression model. Differences between binary dependent variables were examined individually using logit regression with multinomial logit applied to categorical variables and linear regression for mean differences. Regression analysis does not control for sociodemographic differences, as the focus is on the lived experience and population differences, rather than condition-specific effects, potentially offering better insights for clinicians and program planning (14). Differences are reported when the p-value is less than 0.05. Due to the limited sample size of youth with type 2 diabetes (n=45) and the exploratory nature of the study, we did not adjust for multiple comparisons. Adjustment techniques, such as the Bonferroni correction, are exceedingly cautious and can significantly elevate the likelihood of Type II error, especially in small samples, hence potentially masking significant contrasts (15).

IRB: This study was determined to be exempt from regulation as *not human research* by the University of Minnesota Human Subjects Research Protection Program.

## Results

Our analysis identified 43 adolescents (ages 10–17 years) with type 2 diabetes with the prevalence somewhat lower (Wald test, p=0.144) among those aged 10–14 years (0.17%, 95% CI: 0.05%-0.28%) compared to adolescents aged 15–17 years (0.32%, 95% CI: 0.15%-0.48%). Demographic characteristics are detailed in **Table 1**.

**Table 1:** Sample demographic characteristics by youths’ parent reported condition.

	Type 2 Diabetes		Asthma			SHCN			No SHCN, Asthma or Diabetes		
	mean		mean			mean			mean		
Age of Selected Child - In Years (SD)	14.5 (1.86)		13.5 (2.22)	*		13.7 (2.42)	*		13.4 (2.13)	*	
	n	Weighted %	n	Weighted %		n	Weighted %		n	Weighted %	
Sex of Selected Child											
Male	20	36.0%	1197	51.0%	NS	3109	50.0%	NS	8215	52.0%	NS
Female	23	64.0%	1019	49.0%		3057	50.0%		7533	48.0%	
Race and ethnicity											
Hispanic	10	22.0%	409	29.0%	*	803	23.0%	*	2625	29.0%	*
White, non-Hispanic	13	20.0%	1263	40.0%		4343	54.0%		9749	46.0%	
Black, non-Hispanic	8	28.0%	243	20.0%		349	13.0%		1079	13.0%	
Other/Multi-racial, non-Hispanic	12	30.0%	301	11.0%		671	10.0%		2295	13.0%	
Income based on federal poverty level status											
0-99% FPL	13	29.0%	370	22.0%	NS	796	20.0%	*	2000	17.0%	*
100%-199% FPL	10	29.0%	425	25.0%		1035	21.0%		2525	20.0%	
200%-399% FPL	12	24.0%	605	28.0%		1751	26.0%		4575	29.0%	
400% FPL or greater	8	18.0%	816	25.0%		2584	32.0%		6648	34.0%	

* indicates difference from the T2D group is significant at p<.05 for the mean age, and the distribution of race/ethnicity and poverty level.

NS indicates difference from the T2D group is NOT significant at p<.05.

Data from the 2022 National Survey of Children’s Health.

SHCN, Special Healthcare Needs.

Parents of adolescents with type 2 diabetes differed from one or more groups in the following: poor coping, feelings of aggravation towards their adolescent, and extent of family resilience. (**Table 2**).

**Table 2:** Parent and family strengths and challenges.

	Type 2 Diabetes		Asthma			SHCN			No SHCN, Asthma or Diabetes		
	n	Weighted %	n	Weighted %		n	Weighted %		n	Weighted %	
Family share ideas/talk about things that matter											
Somewhat/not very well	23	45.0%	934	42.1%	NS	3,272	55.2%	NS	5,742	36.7%	NS
Very well	19	55.0%	1,249	57.9%		2,814	44.8%		9,650	63.3%	
Family talk about facing problems											
Some or None of the time	10	26.3%	258	12.6%	NS	846	15.7%	NS	1,489	12.0%	NS
All or Most of the time	30	73.7%	1,888	87.4%		5,160	84.3%		13,618	88.0%	
Parent is coping well											
Very well	17	45.7%	1,109	53.6%	NS	2,530	43.8%	NS	8,916	59.7%	NS
Somewhat	20	46.3%	1,026	42.9%		3,311	51.2%		6,341	38.9%	
Not very	5	8.0%	50	3.5%		265	5.0%		205	1.4%	*
Parent aggravation											
No	29	75.5%	2,002	91.2%	*	5,031	80.0%	NS	15,113	97.0%	*
Yes	12	24.5%	181	8.8%		1,067	20.0%		315	3.0%	
Parent has emotional support											
No	15	36.7%	468	26.2%	NS	1,303	24.0%	NS	3,451	27.9%	NS
Yes	25	63.3%	1,703	73.8%		4,785	76.0%		11,884	72.1%	
Family demonstrates qualities of resilience											
All or most of the time to 0–1 items	6	24.0%	138	7.6%	NS	570	10.4%	NS	803	6.3%	NS
All or most of the time to 2–3 items	7	7.0%	257	12.2%		780	12.6%		1,404	11.0%	
All or most of the time to all 4	27	69.0%	1,756	80.2%		4,671	77.0%		12,928	82.7%	

*indicates difference from the T2D group is significant at p<.05 for binary variables and for the distribution of responses when there is greater than two categories.

NS indicates difference from the T2D group is NOT significant at p<.05.

Data from the National Survey of Children’s Health 2022.

SHCN, Special Healthcare Needs.

Adolescents with type 2 diabetes were reported to have poorer health, higher rates of depression and anxiety, lower flourishing scores and higher hospitalization (25.7%) and emergency department (ED) use (33.0%) than those in one or more of the other groups. (**Table 3**). Analyses testing for differences between groups controlling for socio-demographic characteristics is available from the authors.

**Table 3:** Youth health and health services use.

	Type 2 Diabetes		Asthma			SHCN			No SHCN, Asthma or Diabetes		
	n	Weighted %	n	Weighted %		n	Weighted %		n	Weighted %	
Youth General Health											
Fair or Poor	15	31.8%	113	4.8%	*	218	4.0%	*	68	0.6%	*
Excellent, V. Good or Good	28	68.2%	2,101	95.2%		5,928	96.0%		15,637	99.4%	
Youth has Depression											
No	30	71.5%	1,820	86.7%	*	4,431	74.7%	NS	15,398	98.7%	*
Yes	12	28.5%	381	13.3%		1,672	25.3%		254	1.3%	
Youth has Anxiety											
No	31	77.6%	1,529	76.2%	NS	3,127	54.9%	*	14,740	95.3%	*
Yes	11	22.4%	653	23.8%		2,931	45.1%		883	4.7%	
Child is Flourishing (stay calms, shows interest, finishes tasks)											
No	28	46.8%	1,061	46.7%	NS	3,999	67.1%	*	4,787	31.9%	NS
Yes	15	53.2%	1,136	53.3%		2,126	32.9%		10,761	68.1%	
Youth had an ER Visit in Past Year											
No	31	67.0%	1,728	78.5%	NS	4,975	78.2%	NS	14,111	89.1%	*
Yes	12	33.0%	480	21.5%		1,178	21.8%		1,548	10.9%	
Youth had a Hospital Stay in Past Year											
No	34	74.3%	2,118	96.2%	*	5,789	93.2%	*	15,460	98.8%	*
Yes	9	25.7%	85	3.8%		358	6.8%		181	1.2%	
Youth had a Preventive Care Visit in Past Year											
No	8	11.5%	351	18.6%	NS	808	14.7%	NS	4,490	32.8%	*
Yes	34	88.5%	1,832	81.4%		5,279	85.3%		11,015	67.2%	

*indicates difference from the T2D group is significant at p<.05.

NS indicates difference from the T2D group is NOT significant at p<.05.

Data from the National Survey of Children’s Health 2022.

SHCN, Special Healthcare Needs.

## Discussion

Parents reported high psychosocial stress, aggravation, and low resilience when compared to parents of children without chronic illness, which can significantly impact functioning (16). Consistent with the literature, caregivers of adolescents with type 2 diabetes reported significantly greater mental health concerns, specifically higher rates of depression, as compared to caregivers of youth without a chronic condition, as well as caregivers of youth with asthma. Despite more regular healthcare appointments, they also indicated greater use of emergency and hospital services, which is particularly concerning for adherence and transition to more independent adult care.

These findings underline the importance of psychosocial support not only for pediatric patients with type 2 diabetes, but for their families. Psychosocial interventions have been found to be helpful for children and families across a variety of pediatric chronic illnesses (17, 18). The literature on pediatric weight management emphasizes the importance of a family-based approach for lifestyle change that provides support to parents in addition to the identified patient (19). While there is often overlap in weight management and type 2 diabetes care, there are also unique challenges associated with type 2 diabetes (e.g., insulin management, diabetes stigma) that require specific intervention strategies. Despite the need for specific intervention, few studies have examined psychosocial intervention for families and adolescents with type 2 diabetes (20, 21), with previous studies primarily examining behavioral lifestyle modification without specific parenting support. Thus, parenting support in type 2 diabetes continues to be an area of need (22).

This study highlights the unique challenges associated with type 2 diabetes for patients and families regarding parenting challenges, resilience, and child flourishing. The study presents several limitations, including reliance on self-reported survey data, which opens the possibility of bias and misreporting of healthcare data. The response rate is also low, which can leave the possibility of response bias. The sample size for type 2 diabetes is also small and questions were limited to those available on the standardized survey, which does not allow for more refined understanding of variable studied. Another limitation is the use of multiple comparisons, which may increase the likelihood of Type I error. Despite these limitations, this study also provides a unique opportunity to examine the impact of type 2 diabetes for pediatric patients and their families using a nationally representative group. Further investigation of stressors for children with type 2 diabetes and their families using a larger sample size is required in future studies. Future directions should also examine targeted psychosocial intervention for children and their families with type 2 diabetes, including child and family behavioral health interventions, mental health supports for parents due to stressors associated with parenting a child with a chronic health condition, and targeted social work supports (e.g., financial support for healthier foods, access to transportation to appointments). These could also include eHealth interventions, which have demonstrated efficacy, while reducing the burden of multiple appointments on families (23). Thus, future intervention studies for parenting, psychosocial, and lifestyle stressors associated with type 2 diabetes are necessary.

## Data Availability

The original contributions presented in the study are included in the article/supplementary material. Further inquiries can be directed to the corresponding author. The dataset, including constructed variables, are also publicly available to download at https://www.childhealthdata.org/.
